# Knee Joint Distraction for Bicompartmental Knee Osteoarthritis in Asian Patients

**DOI:** 10.5704/MOJ.2511.005

**Published:** 2025-11

**Authors:** WSR Lim, J Soong, DTS Koh, HR Bin-Abd-Razak, KH Lee

**Affiliations:** Department of Orthopaedic Surgery, Singapore General Hospital, Singapore

**Keywords:** knee joint distraction, osteoarthritis, joint-preservation

## Abstract

**Introduction:**

Young active patients with significant pain from knee osteoarthritis are a challenging group of patients to treat. For patients with symptomatic osteoarthritis involving both medial and lateral compartments, total knee arthroplasty (TKA) would traditionally be their only surgical option. Knee joint distraction (KJD) is a novel procedure in Asia that offers a joint preserving alternative for this cohort of patients. This study aims to evaluate patients with knee osteoarthritis treated with knee joint distraction (KJD).

**Material and Methods:**

Patients were included in this study if they had medial and lateral knee pain refractory to conservative treatment for more than 6 months, aged less than 50 and radiographs confirmed osteoarthritic changes in both the medial and lateral tibio-femoral compartments. An external fixator was placed in the distal femur and proximal tibia, and the knee joint was progressively distracted over a period of 3 days, to a total distance of 5mm. After six weeks, the external fixator is removed. Manipulation under anaesthesia was performed for patients who experienced stiffness post external fixator removal to achieved desired range of motion.

**Results:**

A total of three patients underwent KJD from 2020 to 2021. The patients’ age ranged from 44 to 49 years. The mean pre-operative Oxford Knee Score (OKS) was 37.6. At final follow-up at 2 years, the mean post-operative OKS was 17.6. All patients managed to attain the minimal clinically important difference in the OKS.

**Conclusion:**

In young patients with symptomatic bicompartmental knee osteoarthritis, KJD can be considered before doing a total knee replacement.

## Introduction

Knee osteoarthritis (OA) is a common multi-factorial degenerative joint disease that is characterised by progressive chronic pain and functional disability^1^. As in many cases of degenerative joint diseases, the loss of chondro-protective mechanisms and excessive mechanical loading on the knee leads to the pathological course of the disease. Although knee OA mostly affects older people, the number of young patients seeking medical consultation for symptoms relating to osteoarthritis (OA) of the knee is increasing globally^2^.

Young active patients with significant pain and disability from knee osteoarthritis are a challenging group of patients to treat. As compared to the elderly, these patients participate in more active lifestyle. While they are usually at working age, they tend to have higher expectation with higher physical demand. Moreover, most of these patients who suffered from knee osteoarthritis at a younger age typically have risk factors such as obesity, history of traumatic injuries involving the knee. Joint preservation surgery such as knee osteotomy or unicompartmental knee replacement can provide good outcome if their symptoms are isolated to a single compartment of the knee. Patients with symptomatic osteoarthritis of both the medial and lateral compartment of the knee typically undergo total knee arthroplasty (TKA). However, TKA carries a lifetime revision risk in young patients. Walker-Santiago *et al* reported young patients aged 55-year-old are twice more likely to undergo aseptic loosening requiring early re-operation and component revision, as compared to traditional-aged TKA patients (from 60 to 75 year old)^3^.

In recent developments, Knee Joint Distraction (KJD) has been introduced as part of the armamentarium of knee preservation surgeries. Joint distraction has been first described in 1994 in the treatment of hip joint^4^ which was followed by ankle, knee and small joints of the foot and hand. The technique of KJD involves the temporarily separation of the knee joint with the use of distraction device that is externally applied to distal femur and proximal tibia via bone pins. The early results of KJD in Western nations were promising with improvement in clinical and functional outcomes, as well as evidence of cartilage regeneration^5^. The favourable results with KJD demonstrate its potential as a viable option for management of knee OA to postpone the need for primary TKA and resultantly, reducing the need for future revision knee arthroplasty.

Our study aims to evaluate the mid-term outcomes of Asian patients undergoing KJD for bicompartmental knee osteoarthritis.

## Materials and Methods

Patients from 2020 to 2021 were included in this study if they had medial and lateral knee pain refractory to conservative treatment for more than 6 months, aged less than 50 and radiographs confirmed osteoarthritic changes in both the medial and lateral tibio-femoral compartments. Patients were excluded if they had a positive patella grind test indicating clinically symptomatic patellofemoral arthritis, or inflammatory arthritis or prior history of joint infection.

**Fig. 1:**
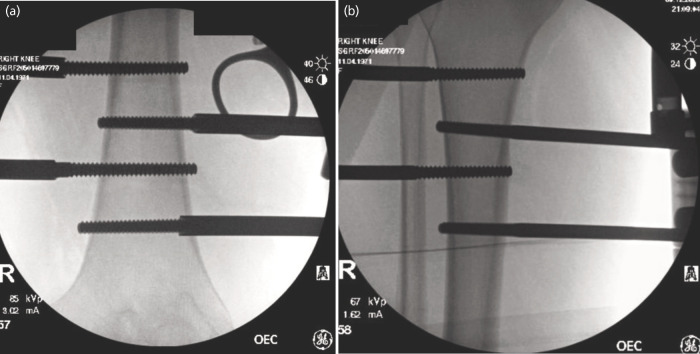
(a) Distal femoral pins application. (b) Proximal tibial pins application intra-operative fluoroscopic imaging.

**Fig. 2:**
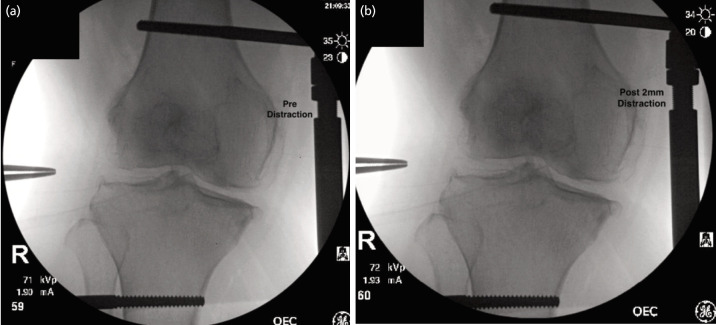
Intra-operative fluoroscopic images (a) pre-distraction and (b) post-distraction of 2mm.

**Fig. 3:**
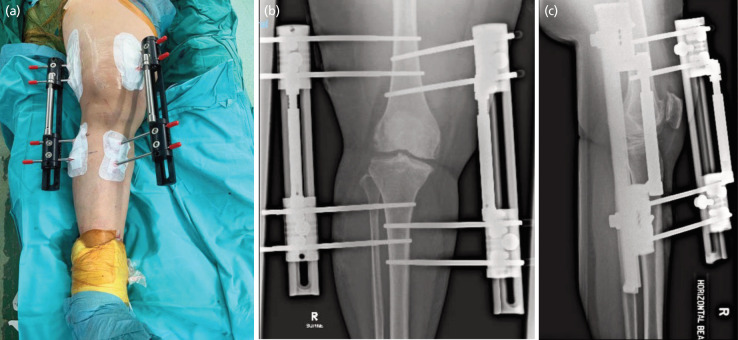
Clinical photographs and post-operative radiographs of knee distraction. (a) Post-application of KJD device. (b and c) KJD with external fixators.

The KJD procedure was performed with the patient on supine position under either general or regional anaesthesia. It involves the use of two monotube distraction device from Limb Reconstruction System [Orthofix®, Texas, United States] applied on either side of the operated knee. The monotubes are anchored to 6mm pins on the distal femur and proximal tibia (Fig. 1). As the distracting forces will be transmitted to the bone-pin interfaces, four bone pins are applied percutaneously in an interdigitating configuration on the respective bones to fortify the construct. The pins were applied extra-capsular, with the most distal femur pin 2cm above the superior pole of the patella, and the most proximal tibia pin 3 - 4cm below the tibial tuberosity. Subsequent pins were spaced 2cm apart from each other. Initial knee joint distraction of 2mm was achieved intra-operatively (Fig. 2). Subsequently, the knee joint was distracted further over a period of 3 days (1mm per day), to a total of 5mm (Fig. 3).

Recent studies have shown evidence of hyaline cartilage repair in KJD. A study by Wiegant *et al* performed biochemical analysis with collagen type II synthesis marker (PIIANP) and collagen type II breakdown marker CTXII and reported an increase ratio of collagen type II synthesis activity over breakdown at up to two years follow-up^7^. Sanjurjo-Rodriguez *et al* analysed the synovial fluids in nine patients who underwent KJD. They reported an increase in the multipotent stromal cells colonies sizes, transcript upregulation of key cartilage core protein aggrecan (ACAN), and significant increase in chondrogenic commitment markers gremlin1 (GREM1) and growth differentiation factor 5 (GDF5)^8^. With more studies in the literature, we can have a clearer understanding on the molecular basis on the role of KJD in intrinsic cartilage healing.

Throughout the distraction phase, patients were allowed to partial weight-bear as tolerated. This is to enable circulation of the synovial fluid with delivery of cytokines and nutrients to the cartilage. To minimise pin track infection, prophylactical antibiotics were given perioperatively. Sterile absorptive foam dressing was applied on the pin sites and changed regularly to remove any exudates or discharge. After six weeks, patients underwent removal of the KJD device and manipulation of the knee under anaesthesia to improve range of motion.

Patients pre- and post-operative range of motion, Oxford Knee Score (OKS) and Knee Society Score (KSS) were evaluated by independent trained personnel. Standing radiographs of the knee and lower limb were taken pre-operatively and post-operatively at six months and two years.

## Results

There were three patients who fulfilled the study criteria and underwent KJD during the study period. Case 1 was a 48-year-old male driver with no medical history but had left knee pain with effusion for several years that was worse when walking on flat ground. Aspiration of the effusion had been done, which was negative for crystals and bacterial growth.

Case 2 was a 49-year-old female waitress who had severe right knee pain when walking and had difficulty standing for extended periods of time. This knee pain had troubled her for several years and was affecting her work.

Case 3 was a 44-year-old male lifeguard with a history of diabetes mellitus and right anterior cruciate ligament reconstruction more than 20 years ago. He complained of chronic right knee pain with that limited his ability to run but had no symptoms of instability. The detailed demographics of the patients are detailed in Table I.

**Table I:** Clinical and radiological outcome of surgery.

Patient Demographics		Case 1	Case 2	Case 3
Age		48	49	44
Gender		Male	Female	Male
Weight (kg)		86	76	84
BMI		31.2	32.4	28.0
**Clinical Outcome**				
Knee Extension (°)	Pre-op	5	10	0
	After removal of distractor	5	0	5
	Post-op 2 years	4	10	2
Knee Flexion (°)	Pre-op	120	105	130
	After removal of distractor	85	40	110
	Post-op 2 years	130	105	142
Oxford Knee Score*	Pre-op	48	40	25
	Post-op	16	19	18
Knee Society Score#	Pre-op	18	18	80
	Post-op	78	78	100
**Radiological Outcome**				
Hip-Knee-Ankle Angle (° varus)	Pre-op	10	8	2
	Post-op 2 years	5	5	2
Joint Width	Pre-op	0 / 5.5	2.1 / 7.2	6.0 / 7.4
(medial/lateral in mm)	Post-op 2 years	4.5 / 4.6	4.0 / 7.6	6.2 / 8.1

Notes - * Lower scores indicate better function, # Higher score indicate better function

The mean pre-operative OKS and KSS was 37.6 and 38.7, respectively. At final follow-up at 2 years, the mean postoperative OKS and KSS was 17.6 and 85.3, respectively. All three patients had improvement in their functional outcome. None of the patients had poorer knee range of motion at two years (Table I).

**Fig. 4:**
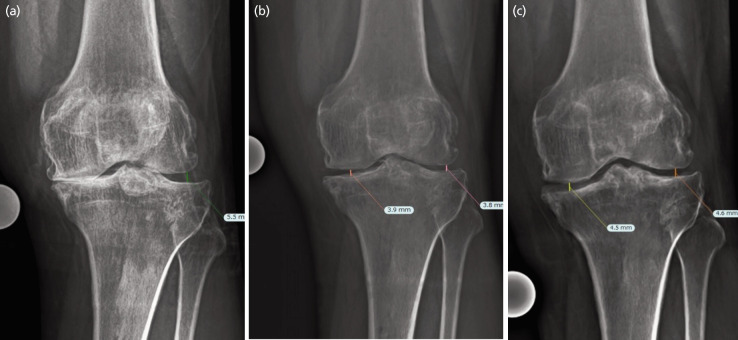
(a) pre-operative, (b) post-operative in six months, and (c) post-operative in two years of weightbearing knee radiograph series showing improvement in the joint width.

**Fig. 5:**
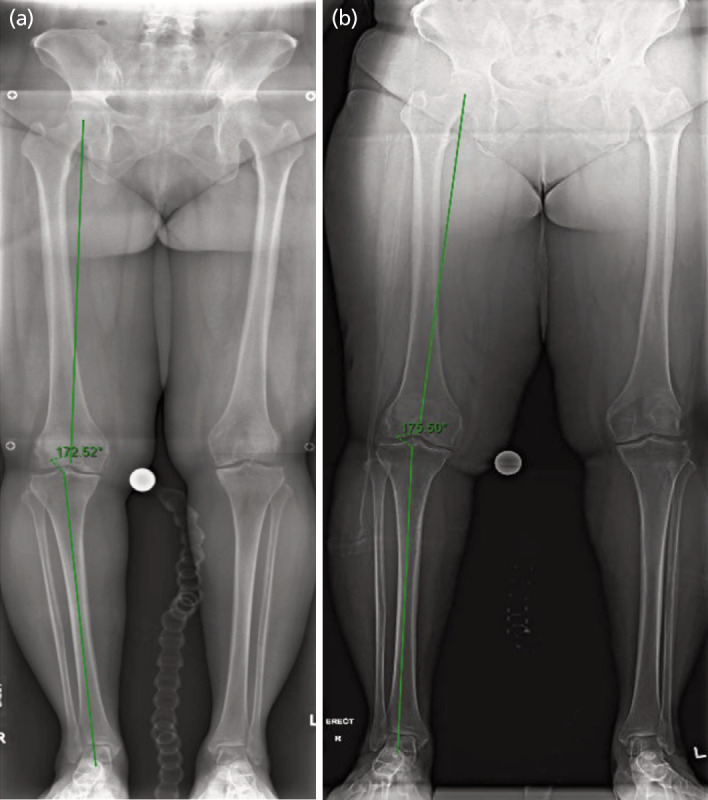
(a) pre-operative, and (b) post-operative of weightbearing lower limb film showing improvement in the HKA angle.

The radiological measurement of the hip-knee-ankle (HKA) alignment and the weightbearing joint width in medial and lateral compartments were performed using TraumaCad [Brainlab AG, German] with a calibrated ball marker. Two out of three patients had improvement in the varus alignment (Table I). It was notable that there was significant improvement in the post-operative radiographs (Fig. 4 and Fig. 5).

There was a single pin tract infection in Case 1, which was successfully treated with antibiotics and did not require surgical debridement.

## Discussion

Osteoarthritis is a biochemically mediated, mechanically driven process which causes debilitating pain and activity limitation in some patients. Knee joint distraction allows complete unloading of the joint which may positively change cartilage metabolism, potentially normalising overall chondrocyte function^1^ and provide an environment favourable for the regeneration of articular cartilage^2^. It has been postulated that weightbearing during the distraction phase causes fluid pressure changes in the joint which may stimulate cartilage matrix synthesis and decrease production of inflammatory cytokines with tumour necrosis factor-alpha and interlukin-16.

Intema first described the use of KJD in the treatment of knee osteoarthritis^5^. They used an external fixator to distract the joint 5mm for 2 months and reported significant improvement in WOMAC score from 45 to 77 as well as a significant increase in cartilage thickness on MRI. Van der Woude *et al* also reported sustained clinical improvement in the WOMAC score at 5 years follow-up in patients undergoing KJD, with a mean increase of 21.1 in the total WOMAC score from baseline and only 3 out of 18 patients converting to a TKA during that period^9^. In our study, none of the patients required to undergo TKA within two years.

Two randomised controlled trials were conducted in Netherlands, one comparing KJD to TKA and another comparing KJD to HTO^10^. At one year follow-up, KJD was not inferior to TKA. However, at two years follow-up, the TKA group showed significantly greater improvement in the WOMAC score compared to KJD. The trial comparing KJD to HTO showed no significant difference in WOMAC score between the two groups at one^11^ or two years^10^.

Abd Razak *et al* proposed several indications for KJD – patients younger than 65 years of age, tibio-femoral pain resistant to conservative treatment, Kellgren and Lawrence Grade 2 – 4 osteoarthritis, end-stage tibiofemoral osteoarthritis and asymptomatic patellofemoral joint osteoarthritis^12^. Since KJD has inferior clinical outcomes to TKA at 2 years, it might be better to restrict KJD to patients for whom TKA has an unacceptably high lifetime revision risk and burden, such as men in their early fifties, whose risk range from 25% to 35%^13,14^. In such patients, KJD can be a temporising procedure to relief arthritic pain before TKA is required at a later age. Eventual conversion to primary TKA at a later age should not be viewed upon as a failure of the procedure but rather a natural progression of the osteoarthritic process. As these patients undergo primary TKA at an older age, the likelihood of requiring a revision TKA in their lifetime would be reduced.

While KJD may be a promising solution to young patients suffering from knee osteoarthritis, known complications such as pin tract infection, nerve and vascular injury from pin insertion and thromboembolic events may occur^12^. Jansen reported 55% of patients undergoing KJD experienced pin tract infections, the majority of which were successfully treated with oral antibiotics^10^. In our cases, we had one patient who had a superficial infection of one of the pin sites, which resolved with topical and oral antibiotics. The pins provide a communication between the external environment and the bones they are placed in; thus, it is unsurprising that risk of infection in KJD is significantly higher compared to TKA or HTO^15^. Given the high rate of pin tract infections, there have been concerns if this may increase the risk of prosthetic joint infections should TKA be necessary in the future^16^. In five patients who underwent TKA after KJD, Wiegant reported that complications such as delayed wound healing and superficial wound infection^17^. The authors believe that one possible improvement in KJD technique would be an internalised distraction device to mitigate the issue of pin tract infection. A hinged, internalised KJD device may also be designed to allow safe ranging of the knee during the distraction phase but will also require a second surgery for removal of implants.

## Conclusion

Our study has demonstrated that with the correct patient selection and correct indication, KJD provides reliable pain relief in patients with bicompartmental knee osteoarthritis in Asian patients.
